# Analysis of immune-related genes during Nora virus infection of *Drosophila melanogaster* using next generation sequencing

**DOI:** 10.3934/microbiol.2018.1.123

**Published:** 2018-02-27

**Authors:** Wilfredo Lopez, Alexis M. Page, Darby J. Carlson, Brad L. Ericson, Matyas F. Cserhati, Chittibabu Guda, Kimberly A. Carlson

**Affiliations:** 1 Biology Department, University of Nebraska at Kearney, Kearney, NE 68849, USA; 2 Department of Genetics, Cell Biology and Anatomy, University of Nebraska Medical Center, Omaha, NE 68198, USA

**Keywords:** Nora virus, immunity, *Drosophila melanogaster*, RNA seq, next generation sequencing, immune-related genes

## Abstract

*Drosophila melanogaster* depends upon the innate immune system to regulate and combat viral infection. This is a complex, yet widely conserved process that involves a number of immune pathways and gene interactions. In addition, expression of genes involved in immunity are differentially regulated as the organism ages. This is particularly true for viruses that demonstrate chronic infection, as is seen with Nora virus. Nora virus is a persistent non-pathogenic virus that replicates in a horizontal manner in *D. melanogaster*. The genes involved in the regulation of the immune response to Nora virus infection are largely unknown. In addition, the temporal response of immune response genes as a result of infection has not been examined. In this study, *D. melanogaster* either infected with Nora virus or left uninfected were aged for 2, 10, 20 and 30 days. The RNA from these samples was analyzed by next generation sequencing (NGS) and the resulting immune-related genes evaluated by utilizing both the PANTHER and DAVID databases, as well as comparison to lists of immune related genes and FlyBase. The data demonstrate that Nora virus infected *D. melanogaster* exhibit an increase in immune related gene expression over time. In addition, at day 30, the data demonstrate that a persistent immune response may occur leading to an upregulation of specific immune response genes. These results demonstrate the utility of NGS in determining the potential immune system genes involved in Nora virus replication, chronic infection and involvement of antiviral pathways.

## 1. Introduction

Nora virus is a picorna-like virus that infects various *Drosophila* species and produces a non-pathogenic persistent infection [1],[2]. The single-stranded, positive-sense RNA viral genome is approximately 12 kilobases (kb) and encodes four open readings frames (ORFs) [1], unlike other picorna-like viruses such as *Drosophila* C virus, which only has two ORFs [3]. ORF1 encodes an RNA interference (RNAi) suppressor and suggests this may contribute to the establishment of persistent infection. In addition, other known innate immune pathways are not involved in regulation of infection [4]. ORF2 encodes a replicative cassette, which consists of a helicase, protease and RNA-dependent RNA polymerase [1]. ORF3 encodes VP3 and is essential for the stability of the viral capsid during environmental stress, such as heat or protease exposure [5],[6]. ORF4 encodes VP4, which is processed into three major proteins: VP4A, VP4B and VP4C [7]. Viral localization, replication, and assembly is hypothesized to occur in the gut and transmitted fecal-orally with no exhibited pathology in gut tissue [8]. Gene expression due to viral infection, as well as control of gene regulation due to viral replication, spread and chronic infection, is not well understood. Interactions of the virus with the *Drosophila* immune system are essential in understanding these processes. Major mechanisms of innate immunity include the immune deficiency pathway (Imd) [9], Toll-Dorsal pathway (Toll) [10], Janus kinase/signal transducer and activator of transcription pathway (JAK/STAT) [11] and RNA interference (RNAi) [12]. Prior research was conducted with cDNA microarray to determine genetic expression due to infection four days post-eclosion of *D. melanogaster*. The results of the study were limited in that only 58 genes were able to be analyzed (46 upregulated and 12 downregulated) and only a small number of these were related to the innate immune response. The main limitation of this study was that it only assessed a single early time point that did not represent the overall time course of infection in *D. melanogaster* [13]. In addition, the technology used in this study is currently outdated and is being “sunsetted” in many institutions. However, technological advances have provided better tools to analyze in-depth gene expression during various time points of infection. Next generation sequencing (NGS) allows for faster, inexpensive, and more accurate, reliable results than prior sequencing methods. In addition, NGS does not require validation of results via quantitative RT-PCR (qRT-PCR) [14]. In this study, we expanded the sampling days to 2, 10, 20 and 30 post-eclosion to capture a more complete picture of gene expression profile changes over time. From this data, we used NGS to determine differentially expressed genes (DEGs) during Nora virus infection at day 2, 10, 20 and 30, which were time points selected from the data of the time course. The objective of this study was to determine the immune related genes that are regulated by Nora virus infection and how this changes over time. The data from this study can be used to further analyze and determine pathways that may be involved in regulation of infection, viral replication and potential pathogenesis.

## 2. Materials and methods

### 2.1. D. melanogaster husbandry, infection and time course experiment

White-eyed (*w^1118^*) flies (Vienna *Drosophila* Resource Center, Vienna, Austria) were maintained in an incubator at 25 °C on a standard cornmeal, torula yeast, molasses medium with a diurnal light cycle. Flies were infected fecal-orally to establish Nora virus infected and uninfected stock for further analysis [13]. Once adequate stocks were established, stock bottles were expanded for fly collection by transferring flies into new bottles. The next generation of flies were allowed to eclose and virgin female flies were collected for further analysis. Flies were placed in pint cages with air ventilation, a food vial, and an access point for a mouth aspirator to remove and add flies. The following two conditions were established: Nora virus infected flies (NV+) and Nora virus uninfected (NV−) flies. One hundred virgin female flies were added to an individual cage and three cages were set-up per condition for a total of six cages. Flies were collected at 2, 10, 20 and 30 days post-eclosion, placed into a microcentrifuge tube, and frozen at −80 °C. Flies were screened for productive Nora virus infection by RT-PCR. Since *D. melanogaster* are invertebrates, it was not necessary to obtain permission or permits to carry out this research.

### 2.2. RT-PCR and qRT-PCR analysis of Nora virus

Total RNA extraction was performed using TRIzol^®^ per manufacturer's instructions (ThermoFisher Scientific, Waltham, MA). Samples were analyzed for the presence of Nora virus using Nora *ORF1* 55–844 (Forward 5′-TGGTAGTACGCAGGTTGTGGGAAA-3′; Reverse 5′-AAGTGGCATGCTTGGCTTCTCAAC-3′) and Promega Access Quick RT-PCR master mix and reverse transcriptase (Madison, WI) according to manufacturer's instructions. Reactions were set-up in duplicate under the following conditions: 50 °C for 30 min, 94 °C for 2 min (94 °C for 30 s, 55 °C for 30 s, 68 °C for 1 min) for 30 cycles, 68 °C for 5 min, and hold at 4 °C. A positive reaction yielded a product at approximately 790 bp for Nora virus. TaqMan Gene Expression Assay kits (Applied Biosystems, Foster City, CA) and the 7500 Real Time PCR System (Applied Biosystems) were used to perform reverse transcription quantitative PCR (qRT-PCR) according to manufacturer's instructions. The TaqMan probe sets were Ribosomal protein L32 (*RpL32*; endogenous control; assay #Dm02151827_g1) and Nora virus (AIRSA9W). Duplicate reactions for each of 3 experiments (n = 6) were carried out under the following conditions: 48 °C for 30 min, 95 °C for 10 min (95 °C for 15 s, 60 °C for 1 min) repeated for 40 cycles. The PCR products were analyzed in the linear range for amplification with *RpL32* using the 7500 Real Time PCR System Sequence Detection Software^®^ (Applied Biosystems). The relative quantitative results were used to determine changes in gene expression on a Log2 scale via the ΔΔCT method [15]. Statistical analysis was conducted using an unequal variance, two-tail *Student's* t-test.

### 2.3. Next generation sequencing and gene expression analyses

Next generation sequencing was used to compare triplicate samples of NV+ and NV− flies at different time points (2, 10, 20 and 30 days post-eclosion) to determine the best time for significant infection and to assess genes upregulated and downregulated during infection. Data analysis on the NGS results was conducted on RNA extractions of the samples at the UNMC Genomics Core Facility (Omaha, NE) and sequenced using the Illumina HiSeq system. RNA sequencing libraries were constructed using 750 ng of total RNA from each sample and the TruSeqV2 kit from Illumina (Illumina, San Diego, CA) following manufacturer's protocol. The libraries were subjected to 100 bp single read sequencing using a HiSeq2500 sequencer to generate approximately 12 to 15 million reads per sample. Fastq files were generated using the bc12fastq software, version 1.8.4 and provided to the UNMC Bioinformatics Core facility for further analysis. The fastq files for each sample were analyzed using the Tuxedo pipeline to find differentially expressed genes. Read alignment was done using tophat version 2.0. FPKM values (which measure gene expression levels) were calculated with cufflinks 2.2. The cuffmerge and cuffdiff software were used to calculate fold change values (afterwards re-calculated to log2 fold change values) between sets of samples. A p-value of 0.05 was used to differentiate between statistically significant and insignificant genes. Version dm6 of the *D. melanogaster* annotation was downloaded from the UCSC website: http://hgdownload.soe.ucsc.edu/goldenPath/dm6/bigZips/. Differentially expressed genes [log2 (fold change) log2 ≥ 1 or ≤−1] were subjected to ontology analyses using PANTHER (Protein Analysis through Evolutionary Relationships; http://www.pantherdb.org/; Table 1) [16],[17]. Genes that were not categorized as immune system processes by PANTHER and have a role in the immune response were noted (Supplemental Table 3). Venn diagrams were created using the Venn diagram software at the PSB website at http://bioinformatics.psb.ugent.be/cgi-bin/liste/Venn/calculate_venn.htpl. Up and downregulated gene sets from each comparison were individually uploaded to the DAVID database (https://david.ncifcrf.gov/home.jsp) [18],[19]. Official gene symbols were used, using gene lists, not background lists. Information for functional annotation charts and tables were retrieved. Gene lists for Gene Ontology (GO) terms for immune response (GO: 0006955, immune response and GO: 0045087, innate immune response) were noted, along with the number of genes and the p-value for the specific GO term. Different combinations of immune-related genes for control (Nora virus negative; NV−) versus Nora virus positive (NV+) comparisons were depicted in a Venn diagram for upregulated genes.

## 3. Results

### 3.1. Validation of Nora virus infection by RT-PCR and qRT-PCR

All Nora virus infected samples demonstrated the expected RT-PCR amplification product for Nora virus ORF1 at 790 bp, whereas the uninfected controls did not (data not shown). Quantitative RT-PCR was used to analyze Nora viral load across time points (2, 10, 20 and 30 days post-eclosion). The gene expression data from qRT-PCR was normalized using *RpL32* and reported as average fold change (n = 6) relative to Nora virus free flies. Viral load significantly increased (*p* < 0.05) at all time points over the time course of the experiment. At day 2, the viral load was 2.38 × 10^6^ (*p* = 0.016) and increased to 2.70 × 10^6^ (*p* = 0.014) at day 10. As the infection progressed, the viral load fluctuated, decreasing at day 20 to 6.53 × 10^5^ (*p* = 0.038), and increasing again at day 30 (1.16 × 10^6^; *p* = 0.023). The viral load remained consistent at a power of 10^5^ or higher over the course of the infection. In addition, other Nora virus infected time points were compared with one another and statistical significance was assessed (*p* < 0.05). There was no statistical significance between days 2 and 10, or day 30 compared to days 2, 10 or 20. The only statistical significance in comparing days infected was at day 20, which was significantly reduced compared to day 2 (*p* = 0.013) and day 10 (*p* = 0.048).

### 3.2. Gene expression of Nora virus infection versus uninfected controls

Next generation sequencing was conducted on NV+ and NV− total RNA extractions at day 2, 10, 20 and 30 to evaluate the effects of Nora virus infection on whole genome expression over time. Total RNA extractions for each time point were sequenced and gene expression was evaluated by comparing the sequences to a reference *D. melanogaster* genome. Gene expression was measured by calculating fold change values between sets of samples and assessing statistical significance (*p* < 0.05). An additional cut-off for statistically significant genes was used to determine differentially expressed genes by evaluating fold change (log2 ≥ 1 or ≤−1) and categorized as either upregulated or downregulated. Differentially expressed genes were submitted for classification to the PANTHER database for analysis. The PANTHER Classification system is a method of classifying genes and/or their proteins by functions using experimental evidence and phylogenetic relationships. If functions are not known, predictions can be made based on experimental evidence [15],[16]. Genes whose IDs were recognized were classified and placed into categories with a gene ontology accession number, which includes biological adhesion (GO: 0022610), biological regulation (GO: 0065007), cellular component organization or biogenesis (GO: 0071840), cellular process (GO: 0009987), developmental process (GO: 0032502), growth (GO: 0040007), immune system process (GO: 0002376), localization (GO: 0051179), locomotion (GO: 0040011), metabolic process (GO: 0008152), multicellular organism process (GO: 0032501), reproduction (GO: 0000003), response to stimulus (GO: 0050896), and rhythmic process (GO: 0048511). The number of unrecognized gene IDs was also listed, as well as the number of genes in the GO category from FlyBase [20] (Table 1). The categories that are most largely represented over time are metabolic process (GO: 0008152) and cellular process (GO: 0009987). The least represented categories are growth (GO: 0040007) and rhythmic process (GO: 0048511; Table 1). The number of up and downregulated genes per GO category were used to calculate a p-value as follows. The PANTHER database has information for 13,757 *Drosophila* genes. From the FlyBase database, the number of *Drosophila* genes per GO term were gathered by searching for each GO term (biological process) in the QuickSearch field [20]. Using the number of genes from the up and downregulated gene set for days 2, 10, 20 and 30, a hypergeometric p-value was calculated. All p-values are significant except for only one GO term that is for rhythmic processes (GO: 0048511) (Supplemental Table 1). For NV+ vs. NV− flies, the number of upregulated genes increased for all categories from day 2 to day 10 and day 20 to day 30. A decrease was observed for all categories, from day 10 to day 20. The number of downregulated genes increased for certain categories from day 2 to day 10, biological regulation (GO: 0065007), cellular component organization or biogenesis (GO: 0071840), cellular process (GO: 0009987), localization (GO: 0051179), metabolic process (GO: 0008152), and response to stimulus (GO: 0050896). From day 10 to day 20, an increase was observed for the aforementioned categories as well as locomotion (GO: 0040011), multicellular organism process (GO: 0032501), and reproduction (GO: 0000003). From day 20 to day 30, an increase was observed for the previous categories, biological adhesion (GO: 0022610) and the immune system process (GO: 0002376; Table 1).

**Table 1.** PANTHER analysis of differentially expressed genes across time points for NV+ and NV− flies. The numbers in the column indicate the total number of genes found either upregulated or downregulated for biological processes of each group from the gene identification numbers (IDs) able to be mapped from PANTHER. Some genes may be represented in more than one GO category. *The numbers in the table are unmapped IDs. ^ns^not significant.

Category name (gene ontology accession number)	Day 2		Day 10		Day 20		Day 30		FlyBase [20]
	Up 97*	Down 2*	Up 477*	Down 32*	Up 304*	Down 75*	Up 512*	Down 324*	# Genes in GO Category
Biological adhesion (GO: 0022610)	12	0	30	0	27	0	34	3	669
Biological regulation (GO: 0065007)	78	0	285	7	246	51	312	272	16394
Cellular component organization or biogenesis (GO: 0071840)	30	0	70	10	66	50	85	249	7995
Cellular process (GO: 0009987)	197	0	617	36	513	179	661	783	43160
Developmental process (GO: 0032502)	32	0	136	1	112	10	152	58	5667
Growth (GO: 0040007)	0	0	2	0	1	0	3	1	556
Immune system process (GO: 0002376)	9	0	26	2	21	3	23	8	743
Localization (GO: 0051179)	66	0	168	3	144	22	191	141	12057
Locomotion (GO: 0040011)	6	0	17	0	15	1	21	5	846
Metabolic process (GO: 0008152)	124	0	419	34	340	162	460	701	45390
Multicellular organism process (GO: 0032501)	45	0	208	0	158	7	217	19	8101
Reproduction (GO: 0000003)	4	0	16	0	15	7	21	27	2773
Response to stimulus (GO: 0050896)	48	0	197	8	156	40	173	145	10778
Rhythmic process (GO: 0048511)	6^ns^	0	14	0	5	0	13	0	164

### 3.3. Gene expression evaluation of immune system process (GO: 0002376)

**Figure 1.**
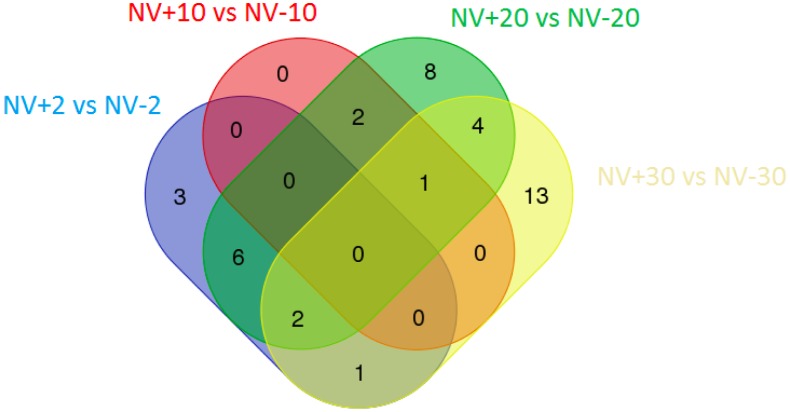
Venn diagram of sets of immune response genes upregulated in Nora virus infected (NV+) and Nora virus uninfected (NV−) flies with comparisons at days 2, 10, 20 and 30. Genes coming from several comparisons (blue: NV+2 vs. NV−2; red: NV+10 vs. NV−10; green: NV+20 vs. NV−20; yellow: NV+30 vs. NV−30) were fed into the DAVID database, and certain immune response-related GO terms were retrieved, which these genes were statistically significantly enriched in (*p* ≤ 5%). The black numbers in the diagram represent the number of immune genes upregulated for that comparison.

To better elucidate the role of the immune response during Nora virus infection, further analysis was conducted for genes associated with the immune system process from the PANTHER analysis. A list was assembled for upregulated and downregulated immune system process genes for NV+ and NV− flies at day 2, 10, 20 and 30. Downregulation of genes only occurred between days 20 and 30 and was associated with genes involved in the immune response. The number of total upregulated immune response genes increased from day 2 to 10 and 20 to 30 and decreased from day 10 to 20 (Supplemental Table 2). Certain immune related genes of interest are *daughter of sevenless* (*dos*), *Dual oxidase* (*Duox*) and *thioredoxin peroxidase 2* (*Jafrac2*), which are all downregulated at day 30 (Supplemental Table 2). Upon further analysis of the data, it was noted that a large number of well-established immune response genes were not identified and genes that are not classified as immune related were determined as such using PANTHER analysis. To further analyze the immune response genes, a list of unique elements was compiled comparing NV+ and NV− flies at days 2, 10, 20 and 30 utilizing the DAVID database. There was a total of 40 unique elements that were upregulated at specific time points during the course of infection (Figure 1). In contrast with what was observed with the total upregulated immune response genes, the number of unique upregulated genes decreased from day 2 to 10, and increased from day 10 to 20, as well as day 20 to 30. At day 2, there were 3 unique genes upregulated: *Drosomycin* (*Drs*), *Spatzle-processing enzyme* (*SPE*), and *Immune induced molecule 14* (*IM14*). There were no immune related genes observed at day 10. When day 20 was analyzed, there were 8 genes that were upregulated. These genes included *Transglutaminase* (*Tg*), *Turandot M* (*TotM*), *Modular serine protease* (*modSP*), *Persephone* (*psh*), *Gram-positive specific serine protease* (*grass*), *Toll-7*, *FER tyrosine kinase* (*FER*) and *Ectoderm-expressed 4* (*Ect4*). At day 30, there were 13 genes identified, which was the largest number of unique genes that were upregulated. This could indicate a specific immune response has developed by day 30. These genes included *eiger* (*egr*), *Necrotic* (*nec*), *Gustatory receptor 28b* (*Gr28b*), *Thor*, *CG42339*, *Secreted Wg-interacting molecule* (*Swim*), *Peptidoglycan recognition protein LA* (*PGRP-LA*), *PGRP-SC1A*, *Scavenger receptor class C type I* (*Sr-CI*), *CG15529*, *Scavenger receptor class C*, *type IV* (*Sr-CIV*), *Activating transcription factor 3* (*Atf3*). In three of the four time points, upregulation was observed in 3 genes: *Toll-2*, *PGRP-SC2*, *Gram-negative bacteria binding protein* (*GNBP1*) (Figure 1).

Once again while observing the data, it was noted that the DAVID analysis also failed to detect well-established immune related genes, especially at day 10. Therefore, the data (Supplemental Table 4) was further characterized by comparing the Nora virus infected versus uninfected flies for days 2, 10, 20 and 30 to a published list of *Drosophila* genes with potential relevance to the immune response (https://lemaitrelab.epfl.ch/page-7767-en.html) compiled by Bruno Lemaitre's lab [21], FlyBase [20], and the Interactive Fly list of RNAi genes [22]. The Lemaitre list contains *Drosophila* genes known to function in immunity, share homology with genes involved in immune reactions in other organisms, and are reported to be induced upon septic injury. The list is largely restricted to genes with high probability protein matches and are also found in FlyBase [20],[21]. In contrast to both PANTHER and DAVID analysis, a large number of immune related genes were found to be differentially regulated over time in Nora infected versus uninfected flies (Table 2; Supplemental Table 3). The immune related genes were separated into different categories dependent upon their function using the list of immune response genes generated by the Lemaitre lab [21]. These categories are microbial recognition and phagocytosis, Toll and IMD pathway signaling, other signaling, melanization, coagulation, hematopoiesis and cellular response, antimicrobial peptides, antiviral defense, stress response and induced molecules. The complete list of genes in each of these categories for each day are listed in Supplemental Table 3. The microbial recognition and phagocytosis category contains a number of subcategories including the peptidoglycan recognition protein (PGRP) family members *PGRP-SC1a*, *SC2*, *LA*, *LB*, *LC*, *SA*, which are all upregulated, whereas *PGRP-LD* are downregulated; the gram negative binding protein (GNBP) family members *GNBP1*, *2*, *3* are up-regulated from days 10 through 30; the complement-like proteins/Thiol Ester Proteins (TEP) that may play a role in the opsonization of microorganisms including *Tep2*, *3*, *4* are upregulated from days 10 to 30; and phagocytosis receptors such as *Down syndrome cell adhesion molecule* (*Dscam*) *1*, *2*, *3*, *4*, of which, *Dscam 1*, *2* are upregulated starting at day 2 through 30, whereas *Dscam 3*, *4* are upregulated at days 10 through 30. The Toll and IMD pathway signaling group contained *nec* upregulated from day 2 through 30; *dorsal related immunity factor* (*dif*) upregulated day 10 through 30; *Toll* receptors *Toll-6* upregulated on days 20 and 30, *Toll-7* upregulated from day 10 through 30, and *Toll-9* upregulated on day 30; *TNF-receptor-associated factor 4* (*Traf4*) and *Traf-like* are upregulated at days 10 and 30; at day 30 *cactus* (*cact*), *spätzle* (*spz*), *tube* (*tub*), *Toll* (*Tl*), *caspar* (*casp*), *Fas-associated death domain* (*Fadd*), *pellino* (*Pli*) are all downregulated at day 30. The other signaling category contained two components of the Eiger (egr)/Wengen (wgn) pathway that have similarities to mammalian TNF and TNFR. The *wgn* gene is upregulated from day 10 through 30, whereas *egr* is upregulated at days 10 and 30, but not day 20. Only one component of the JAK/STAT pathway was detected. At day 30, *hopscotch* (*hop*) is downregulated. The melanization category contained *yellow-f2* and the serine protease *melanization protease 1* (*MP1*) that are both upregulated from days 10 through 30. One other melanization gene, *Chorion protein 19* (*Cp19*), is upregulated from days 2 through 20, but on day 30 it is downregulated. The coagulation category contained five genes that are upregulated from days 2 through 30, including *Hemolectin* (*Hml*), *fondue* (*fon*), *glycoprotein 150* (*Gp150*), *Gelosolin* (*Gel*), and *Apolipophorin* (*Rfabg*). The hematopoiesis and cellular response category contains two genes, *serpent* (*srp*) and *PDGF- and VEGF-receptor related* (*pvr*), that are upregulated from days 2 through 30. In addition, *pvr adaptor protein* (*PVRAP*) and *lozenge* (*lz*) are upregulated from days 10 to 30. The antimicrobial peptides group has two members, *CG16756* and *Nimrod B2* (*NimB2*), which are upregulated from days 2 through 30; three members, *CG16799*, *NimB3*, *NimB5*, are upregulated from days 10 through 30; *Drs* and *Drs-like 5* (*Drsl5*) are upregulated at days 2 and 10; *Listericin*, *NimB4*, and *NimC1* are upregulated at days 10 and 30; *Attacins A* and *Attacins* *B* (*attA*, *B*), *Drsl4* are upregulated at day 10; *Lysozyme X* (*LysX*) and *Diptericin* (*Dpt*) are downregulated at days 10 and 30; *AttD* and *Drsl2* are downregulated at day 30. In the antiviral category, only one gene, *virus-induced RNA 1* (*vir1*) is upregulated from days 2 through 30, whereas this category has the largest number of genes downregulated and this occurred at day 30. The genes that are downregulated at day 30 include *Dicer-1* (*Dcr-1*), *Archipelago* (*ago*), *Argonaute 3* (*AGO3*), *drosha*, *aubergine* (*aub*), *RNA and export factor binding protein 1* (*Ref1*), to list a few. The stress response category included the subcategories for reactive oxygen species (ROS) and stress factors. In the ROS subcategory, *immune-regulated catalase* (*irc*) is upregulated from days 2 through 30, *Nitric oxide synthase* (*Nos*) is upregulated at day 30, and *Dual oxidase* (*Duox*) is downregulated at day 30. In the stress factors category, *Thor* is upregulated days 10 through 30, *TotA* is upregulated at days 10 and 30, *TotM* is upregulated at day 20, and *TotC* is upregulated at day 30. The induced molecules category contains the subcategories induced serine proteases, induced serpins, and induced small molecules, and all of the genes in this category are upregulated. In the serine proteases subcategory, *Ser7*, *Jonah* (*Jon*) *25Biii*, *Jon65Aii*, *Jon65Aiii*, *Jon74E*, *Jon99Ci*, *Jon99Cii*, *Jon99Ciii*, *Jon99Fii* are all upregulated from days 2 through 30; *Jon65Ai* and *Jon99Fi* are upregulated from days 10 through 30; *Jon65Aiv* is upregulated at days 2, 20 and 30. In the induced serpins (spn) subcategory, only *Spn43Ab/Ad* was upregulated at all days; *Spn28Dc*, *Spn42Dc/Dd*, *Spn47C*, *Spn88Eb* are upregulated at days 10 through 30; *Spn31A* and *Spn77Ba* are upregulated at days 10 and 30. In the induced small molecules category, *IM2*, *IM3* and *IM33* are upregulated from days 2 through 30; *IM4* and *IM14* are upregulated at days 2, 20 and 30; *IM1* is upregulated at days 10 and 30 (Supplemental Table 3).

**Table 2.** Manual analysis of differentially expressed immune related genes across time points for NV+ and NV− flies list of *Drosophila* genes with potential relevance to the immune response [21], FlyBase [20], The Interactive Fly list of RNAi genes [22]. Some genes are represented at more than one time point. The numbers in the column indicate the total number of genes found either upregulated or downregulated for each group.

Category name	Day 2		Day 10		Day 20		Day 30	
	Up 47	Down 0	Up 112	Down 0	Up 86	Down 2	Up 130	Down 26
Microbial recognition and phagocytosis	9	0	27	0	22	1	28	2
Toll and IMD pathway signaling	4	0	14	0	10	0	29	0
Other signaling	0	0	1	0	1	0	2	1
Melanization	1	0	4	0	3	0	2	1
Coagulation	5	0	6	0	6	0	7	0
Hematopoiesis and cellular response	2	0	4	0	4	0	4	1
Antimicrobial peptides	4	0	14	2	6	0	10	4
Antiviral defense	1	0	1	0	1	1	1	15
Stress response	2	0	5	0	4	0	8	2
Induced molecules	19	0	36	0	29	0	38	0

## 4. Conclusions

Next generation sequencing was conducted during Nora virus infection to identify immune related genes for further analysis. Genes involved during Nora virus infection were identified by comparison of NV+ and NV− flies aged to day 2, 10, 20 and 30. All NV+ samples demonstrated a positive RT-PCR product at 790 bp and the NV− samples did not show a product, as was expected (data not shown). Viral load was assessed by qRT-PCR and found to increase from day 2 to day 10, decrease on day 20, and increase again at day 30. This bimodal trend was again seen when the differentially expressed genes were submitted to PANTHER analysis for further classification (Table 1), as well as the manual analysis of the immune related genes for total number of genes per day either up or downregulated (Table 2; Supplemental Table 4). In addition, the total upregulated genes for the manually characterized immune related genes (Table 2) followed the same bimodal distribution as was seen with Nora virus load. This suggests that as Nora virus load changes, the genes regulated by this virus, either directly or indirectly, change to match the level of virus in the fly. The kinetics of Nora virus infection are largely unknown. Similar bimodal infection distribution and upregulation of genes is seen in human viruses including influenza A [23] and HIV [24]. In terms of the innate immune response, influenza A was correlated with interferon (IFN) release and found to show a bimodal distribution [23]. This suggests that the bimodal distribution of Nora virus load may be correlated with an interferon-like molecule or other cytokine that follows the same pattern.

PANTHER analysis was used to analyze differentially expressed genes over time as mapped by their GO number. The data demonstrate that the number of genes found for each group is not due to chance and are statistically significant, with the exception of GO: 0048511 (Rhythmic process) at day 2 for the upregulated genes (Supplemental Table 1). This may be due to the small number of genes that are currently assigned to this GO group. To begin looking at these possible associations, the immune system genes (Table 1) were further analyzed (Supplemental Table 2). This analysis revealed 35 unique genes differentially regulated over time, with a number of them not having obvious immune-related function when cross-referencing to FlyBase [20]. This is concerning, but not surprising considering the main goal of PANTHER is to infer gene function from sequence databases by using evolutionary relationships and sparse experimental evidence. The authors state that the most important utility of PANTHER is to accurately infer function in this way [16]. Therefore, the genes should not be discounted as possibly immune related genes, but as targets for future analysis. Of more concern was the lack of well-established immune response genes that were not detected in the PANTHER analysis. Due to the discrepancy in the PANTHER analysis, the data was subjected to DAVID analysis (Figure 1). Additional analysis indicated that upregulation of immune response genes decreased at day 10 and increased between days 10 and 20, as well as days 20 and 30 (Figure 1). Interestingly, day 30 contained the most upregulated genes (13) via DAVID analysis, and the down-regulation of three genes, *dos*, *Duox*, *Jafrac2*, via PANTHER (Table 2) and are listed in FlyBase as having immune-related function [20]. This could indicate that there is a unique immune response that Nora virus infection is inducing by day 30. Of the genes upregulated at day 30 four of them are associated with scavenger receptor activity, *CG42339*, *Swim*, *Sr-CI*, *Sr-CIV*. Scavenger receptor activity is associated with plasmatocytes, the *Drosophila* equivalent of macrophages [25]. This could indicate an increase in the amount of plasmatocytes found within the hemolymph of Nora virus infected flies at day 30. Other genes of interest are *nec* and *PGRP-LA*, which are involved in activation of the Toll and Imd pathways, respectively [26],[27]. This indicates that both pathways are upregulated by day 30 of infection. Also of interest is *Atf3*, which is involved in immune system homeostasis [28]. Upregulation of this gene could indicate an imbalance of the immune system. The fact that anti-viral defenses are downregulated and Toll defenses appear to be increased (Table 2), this may further indicate a dysregulation of the immune system by Nora virus. The last gene of interest that is upregulated at day 30 is *Eiger (egr)*, which is a homolog of tumor necrosis factor (TNF) [29].

Even though the DAVID analysis detected some immune response or related genes, it failed to recognize a number of well-established genes in this group. This was especially concerning considering that the day 10 analysis yielded no upregulated genes, even though the raw data depicts the contrary (Supplemental Table 4). To rectify this problem, a list of immune related genes was compiled using a list of *Drosophila* genes with potential relevance to the immune response compiled by Bruno Lemaitre's lab [21], FlyBase [20] and the Interactive Fly list of RNAi genes [22] (Table 2; Supplemental Table 3). The microbial recognition and phagocytosis category contains a number of genes that function in innate immunity. Recognition of pathogens by the innate immune system relies on conserved microbial surface structures that interact with host recognition proteins. Various peptidoglycan recognition proteins (PGRPs) and gram-negative binding proteins (GNBPs), and Down syndrome cell adhesion molecules (Dscams) were upregulated throughout Nora virus infection (Supplemental Table 3). The PGRPs bind to peptidoglycan found in Gram-positive bacteria to further elicit an immune response. In *Drosophila*, multiple PGRP genes have been identified. In this study, *PGRP-SC1a*, *SC2*, *LA*, *LB*, *LC*, *SA* are all upregulated, whereas *PGRP-LD* is downregulated during Nora virus infection (Supplemental Table 3) [30]. The upregulation of *PGRP* genes is not unique to Nora virus infection, in that, *PGRP-SB1* and *PGRP-SD* were significantly upregulated in *Drosophila* infected with SIGMAV and *PGRP-SA* was upregulated in those infected with DCV [31]. This suggests that PGRPs may be differentially induced based upon their surface binding properties. The GNBPs bind to lipopolysaccharides in Gram-negative bacteria and β-1-3 glucans in fungi [32]. The data demonstrate that *GNBP1*, *2*, *3* are up-regulated from days 10 through 30, supporting the idea that virus can selectively induce these extracellular molecules. *PGRP* and *GNBP* genes must work together to activate the Toll and/or IMD pathways to produce an immune response. For the activation of the Toll pathway, PGRP-SA, PGRP-SD and GNBP1 are required for Gram-positive bacteria and GNBP3 is required for fungi. Our analysis indicates that all of these genes are upregulated at the same time points. For activation of the IMD pathway, PGRP-LC and PGRP-LE must work together [33]. Therefore, the analysis indicates that PGRP-LC is upregulated throughout Nora virus infection. Taken together, these data suggest that the Toll and/or IMD pathways may be induced during Nora virus infection in an uncharacterized fashion. Also present in this category were the Dscams that play a role in direct phagocytosis of pathogens as either a signaling receptor or co-receptor. Dscams may have a potential role as opsonins that target pathogens in the hemolymph and a hemocyte-specific loss of Dscam impairs the efficiency of phagocytic uptake [34]. *Dscam-1*, *2*, *3*, *4* are upregulated during Nora virus infection in this study. The mechanisms underlying hemocyte interaction with virus is not well studied, but suggest a role for these cells during Nora virus infection that should be further evaluated. Once a pathogen is recognized and identified, certain immune pathways are activated to provide an adequate response. In our analysis, components of the Toll pathway were upregulated during infection (Supplemental Table 3). Previous research has suggested the Toll pathway is required for resistance to viral oral infections in *Drosophila* and two transcription factors, *dif* and *dl*, are important for production of antimicrobial peptides [10]. Our analysis indicates significant upregulation of *dif* during Nora virus infection. Prior research using Drosophila X Virus (DXV) found that *dif^1^* mutants were more susceptible to infection than control flies. Mutant flies displayed increased viral titers and succumbed to anoxia-induced death earlier than the control flies [35]. Once again this supports the idea that Nora virus may be activating the Toll pathway through *dif* activation during infection and should be further evaluated. Another component of the Toll pathway is the tumor necrosis factor-receptor associated factor (TRAF) family. The *Drosophila* genome contains three genes for the TRAF family: *Traf4*, *traf6*, *traf-like* [36]. The genes *traf4* and *traf-like* were upregulated during Nora virus infection at day 10 and day 30 (Supplemental Table 3), and *traf4* is implicated in immune response [37]–[39]. In addition, *traf-like* is predicted to be involved in the immune response, protein ubiquitination, and signal transduction [37]. It is activated by egr, TNF, binding to Wengen (wgn), a tumor necrosis factor receptor, which signals additional cellular responses. Previous research has implicated Traf4 as a regulator of the JNK pathway and apoptosis [38]. The JNK pathway is a highly conserved pathway, which signals stress response, cell migration, apoptosis, and immune responses [40]. In addition, traf4 is a component of the Toll/Spz/Pelle signaling complex regulating NF-kB activity. However, the role of traf4 in an immune response to pathogens has not been explored [41]. Intestinal *Vibrio cholera* infection in *D. melanogaster* was explored to determine the role of traf4. Previous research shows that the egr/wgn pathway confers resistance to extracellular pathogens within the hemolymph. So, *Traf4* and *egr* mutants were created to assess susceptibility of *V. cholera* infection. Mutants were found to have increased susceptibility to infection when compared to the controls. The results indicate that the egr/wgn pathway is involved in resistance against intestinal infection by programmed cell death of infected cells. However, this hypothesis was unable to be evaluated [39]. In the data, both *wgn* and *egr* were upregulated at all time points (Supplemental Table 3). The upregulation of *wgn*, *egr*, *Traf4* and previous research with *V. cholera* could indicate that Nora virus, which infects the *Drosophila* gut, may be stimulating the egr/wgn pathway. Further research is required to analyze the gut tissue of Nora virus infected *D. melanogaster* to determine if apoptosis is regulated in response to infection.

Several genes categorized as antimicrobial peptides followed the bimodal trend, which correlates with the qRT-PCR data of Nora virus load over time. Specific antimicrobial peptides that were upregulated across the time points include the *Drosomycin* genes, the *Nimrod* genes, and the *Attacin* genes (Supplemental Table 3). The *Drosomycin* family of genes are antifungal defensins that arose from gene duplication [42]. These genes are under the control of the Toll pathway and can be constitutively expressed by simple activation of the Toll pathway, indicating that these genes could have been expressed by activation of the Toll pathway by another pathogen, not necessarily a fungal infection [43]. The *Nimrod* family of genes is characterized by the presence of NIM repeats, which are closely related to EGF repeats. These proteins are secreted or integral to the plasma membrane and act as receptors for bacterial and apoptotic cell phagocytosis, specifically *Staphylococcus aureus* and *Escherichia coli* [44]. Upregulation of both the *Nimrod* genes and *Drosomycin* indicates that Nora virus infection may contribute to the activation of the Toll pathway, which further supports the conclusions presented previously.

Genes involved in antiviral defense also followed the bimodal distribution until day 30 post-eclosion. At day 30, 15 genes were downregulated unlike the previous three time points where no genes were downregulated at days 2 or 10 and only one gene was downregulated at day 20 (Table 2). The genes that were downregulated at day 30 were observed to be part of the RNAi machinery, such as *Dicer 1*, *Argonaute*, *drosha* (Supplemental Table 3). The RNAi machinery uses small RNAs to target complementary RNA and initiate the silencing of its expression. Nora virus is unaffected by any of the RNAi pathways, indicating that the genes encoding those proteins are likely affected by the virus [4]. NGS confirmed that as the *Drosophila* age, genes related to RNAi are suppressed allowing a persistent infection to occur (Supplemental Table 3). Suppression of RNAi is achieved by the C terminus of open reading frame 1 (ORF1) of Nora virus [45]. Viral protein 1 (VP1) does this by interfering with RNA-induced silencing complex (RISC) activity. Interestingly, the only gene associated with antiviral defense that was upregulated at day 30 was *virus induced RNA 1* (*vir-1*). This gene is strongly induced by viral infection but not bacterial or fungal, and is under the control of the JAK/STAT pathway [46]. The gene *vir-1* requires the JAK kinase encoded by *hopscotch* and the cytokine receptor Domeless to be activated. Upregulation of *vir-1* indicates that Nora virus infection was recognized and the JAK/STAT pathway was activated. However, activation of the JAK/STAT pathway was not enough to overcome the downregulation of the RNAi machinery, which plays a key role in *Drosophila* innate immunity to viruses.

During Nora virus infection, stress is placed within *Drosophila* to respond appropriately. Various stress-related genes were upregulated through infection, such as *Irc* (Supplemental Table 3). The Irc (immune-related catalase) protein is a heme peroxidase involved in antioxidant defense protecting host cells from reactive oxygen species (ROS)-induced damage. During microbial infection, Irc can increase host survival by acting as an antioxidant in the gastrointestinal tract of the fly. Microbes are continually ingested and induce ROS production, so an effective mechanism is required to remove ROS before damage can occur [41]. During Nora virus infection, ROS may be increased in the *Drosophila* gut. Regulation of ROS is also seen by the upregulation of *Nos* and downregulation of *Duox* at day 30. This data suggests induction of oxidative stress upregulating *Irc*, but also the egr/wgn pathway to target the source of the oxidative source. This provides further support that the pathway is involved in a gut antiviral response to Nora virus infection.

Within the induced molecules category, the *Jonah* family of genes was upregulated from days 2 through 30 (Supplemental Table 3). The *Jonah* genes are found to be highly concentrated in the midgut and may act in the regulation of viral infection [47]. The serpin family refers to a group of proteins that share similar action of protease inhibition [48]. Genes throughout this family were upregulated at all time points (Supplemental Table 3). Previously, serpins have been shown to be upregulated in response to fungal or bacterial infection [49]. Upregulation of these could indicate a dysregulation of immune response due to Nora virus infection. The immune induced molecules were also upregulated with at least one from this family being upregulated at each time point (Supplemental Table 3). These peptides are secreted and released into the hemolymph upon Toll activation [50]. Upregulation of these genes indicates that the Toll pathway was activated causing secretion of these molecules in response to viral infection.

Nora virus infection in *D. melanogaster* provides an optimal model to study the complex interactions between virus and the invertebrate immune response. Gene expression analysis throughout the course of Nora virus infection may delineate the role of the gut and the Toll pathway in antiviral defense. In addition, the PANTHER and DAVID databases are useful analytic bioinformatics tools, but may need to be further evaluated as they were unable to select known immunity-related genes. Overall, immune genes of interest were discovered using NGS, which may be further investigated to determine their role in antiviral defense or potential pathogenesis.

## Figures and Tables

**Table S1.** Statistical analysis of PANTHER analysis of differentially expressed genes across time points for NV+ and NV− flies. The numbers in the columns indicate a hypergeometric p-value for the PANTHER analysis for days 2, 10, 20 and 30. *The numbers in the table are unmapped IDs, NA = Not applicable because 0 genes were in this specific category. A value of 0 means that the exponent of the p-value was so high (indicating an extremely low p-value), that the p-value cannot be depicted properly by the operating system.

Category name (gene ontology accession number)	Day 2		Day 10		Day 20		Day 30		FlyBase [1]
	Up 97*	Down 2*	Up 477*	Down 32*	Up 304*	Down 75*	Up 512*	Down 324*	# Genes in GO Category
Biological adhesion (GO: 0022610)	4.64e−07	NA	2.59e−30	NA	1.27e−19	NA	2.22e−31	1.63e−47	669
Biological regulation (GO: 0065007)	0	NA	0	0	0	0	0	0	16394
Cellular component organization or biogenesis (GO: 0071840)	4.71e−242	NA	0	6.70e−33	0	1.28e−113	0	0	7995
Cellular process (GO: 0009987)	0	NA	0	0	0	0	0	0	43160
Developmental process (GO: 0032502)	4.50e−130	NA	0	5.30e−28	0	2.00e−97	0	0	5667
Growth (GO: 0040007)	3.04e−10	NA	2.78e−39	0.023156	6.23e−28	3.72e−09	1.10e−46	2.80e−46	556
Immune system process (GO:0002376)	NA	NA	2.31e−49	NA	2.37e−37	NA	8.21e−52	4.96e−42	743
Localization (GO: 0051179)	0	NA	0	8.55e−109	0	0	0	0	12057
Locomotion (GO: 0040011)	7.46e−15	NA	1.05e−56	NA	1.44e−39	5.79e−13	2.74e−58	2.30e−58	846
Metabolic process (GO: 0008152)	0	NA	0	0	0	0	0	0	45390
Multicellular organism process (GO: 0032501)	9.58e−226	NA	0	NA	0	4.77e−179	0	0	8101
Reproduction (GO: 0000003)	1.18e−69	NA	1.58e−257	NA	1.43e−-183	1.50e−38	4.71e−272	6.57e−191	2773
Response to stimulus (GO: 0050896)	0	NA	0	1.19e−68	0	1.02e−256	0	0	10778
Rhythmic process (GO: 0048511)	0.087659	NA	7.51e−05	NA	8.45e−07	NA	5.34e−06	NA	164

**Table S2.** PANTHER analysis of differentially expressed genes across time points for NV+ vs. NV− flies. The numbers in the column indicate the total number of genes found either upregulated or downregulated for the immune system processes that were able to be mapped from PANTHER. *This gene is an orthologue described by PANTHER databases.

Day 2		Day 10		Day 20		Day 30	
Up 9	Down 0	Up 26	Down 2	Up 21	Down 3	Up 23	Down 8
*CG11319*		*Aplip1*	*CG5568*	*Aplip1*	*CG5568*	*Aplip1*	*CG3394*
*CG11407*		*CG11319*	*Hsp27*	*CG11319*	*Hsp26*	*CG11319*	*CG5568*
CG11407*		*CG11391*		*CG11407*	*Hsp27*	*CG11407*	*CG5708*
*CG4461*		*CG11407*		CG11407*		CG11407*	*dos*
*CG45002*		CG11407*		*CG11453*		*CG11453*	*Duox*
*Fatp*		*CG11453*		*CG15611*		*CG15611*	*Hsp26*
*hig*		*CG15611*		*CG30456*		*CG17999*	*Hsp27*
*Mlp60A*		*CG17684*		*CG42673*		*CG30456*	*Jafrac2*
*wry*		*CG17999*		*CG4328*		*CG42673*	
		*CG30456*		*CG4461*		*CG4328*	
		*CG42673*		*CG45002*		*CG4461*	
		*CG4328*		*CG8834*		*CG45002*	
		*CG4461*		*CG9993*		*CG4830*	
		*CG45002*		*Fatp*		*CG8834*	
		*CG7409*		*fw*		*CG9993*	
		*CG8834*		*hig*		*Fatp*	
		*CG9993*		*l(2)efl*		*fw*	
		*Fatp*		Lim3*		*hig*	
		*fw*		*Lim3,Mlp84B*		*l(2)efl*	
		*hig*		*Mlp60A*		Lim3*	
		*Hsp67Bc*		*wry*		*Lim3,Mlp84B*	
		*l(2)efl*				*Mlp60A*	
		Lim3*				*wry*	
		*Lim3,Mlp84B*					
		*Mlp60A*					
		*wry*					

**Table S3.** Gene list of differentially expressed immune related genes across time points for NV+ and NV− flies from the manual analysis using a list of *Drosophila* genes with potential relevance to the immune response [2], FlyBase [1], The Inter*active* Fly list of RNAi genes [3]. The red highlighted genes represent the upregulated genes, whereas the green highlighted genes represent the downregulated genes for each comparison.

**Table S4.** The excel file depicts the data for Nora virus infected (NV+) or uninfected (NV−) flies for each time point (10, 20 and 30) compared to the control time point (day 2). These are comparisons NV+ day 2 vs. day 10, 20 and 30. The comparisons for NV+ vs. NV− for each time point (2, 10, 20 and 30) are also given. The red highlighted genes represent the upregulated genes, whereas the green highlighted genes represent the downregulated genes for each comparison.

## References

[1] Habayeb MS, Ekengren SK, Hultmark D (2006). Nora virus, a persistent virus in *Drosophila*, defines a new picorna-like virus family. J Gen Virol.

[2] Webster CL, Waldron FM, Robertson S (2015). The discovery, distribution, and evolution of viruses associated with Drosophila melanogaster. PLoS Biol.

[3] Johnson KN, Christian PD (1998). The novel genome organization of the insect picorna-like virus *Drosophila* C virus suggests this virus belongs to a previously undescribed virus family. J Gen Virol.

[4] Habayeb MS, Jens-Ola E, Dan H (2009). Nora virus persistent infections are not affected by the RNAi machinery. PLoS One.

[5] Ericson BL, Carlson DJ, Carlson KA (2016). Characterization of Nora virus structural proteins via Western blot analysis. Scientifica.

[6] Sadanandan SA, Ekström JO, Jonna VR (2016). VP3 is crucial for the stability of Nora virus virions. Virus Res.

[7] Ekström JO, Habayeb MS, Srivastava V (2011). *Drosophila* Nora virus capsid proteins differ from those of other picorna-like viruses. Virus Res.

[8] Habayeb MS, Cantera R, Casanova G (2009). The *Drosophila* Nora virus is an enteric virus, transmitted via feces. J Invertebr Pathol.

[9] De Gregorio E, Spellman PT, Tzou P (2002). The Toll and Imd pathways are the major regulators of the immune response in *Drosophila*. EMBO J.

[10] Ferreira AG, Naylor H, Esteves SS (2014). The Toll-dorsal pathway is required for resistance to viral oral infection in *Drosophila*. PLoS Pathog.

[11] Baeg GH, Zhou R, Perrimon N (2005). Genome-wide RNAi analysis of JAK/STAT signaling components in *Drosophila*. Gene Dev.

[12] Zambon RA, Vakharia VN, Wu LP (2006). RNAi is an antiviral immune response against a dsRNA virus in *Drosophila*
*melanogaster*. Cell Microbiol.

[13] Cordes EJ, Licking-Murray KD, Carlson KA (2013). Differential gene expression related to Nora virus infection of *Drosophila melanogaster*. Virus Res.

[14] Corney DC (2012). RNA-seq using next generation sequencing. Mater Method.

[15] Livak KJ, Schmittgen TD (2001). Analysis of relative gene expression data using real-time quantitative PCR and the 2(-Delta Delta C(T)) method. Methods.

[16] Mi H, Huang X, Muruganujan A (2017). PANTHER version 11: Expanded annotation data from Gene Ontology and Reactome pathways, and data analysis tool enhancements. Nucleic Acids Res.

[17] Thomas PD, Kejariwal A, Guo N (2006). Applications for protein sequence-function evolution data: mRNA/protein expression analysis and coding SNP scoring tools. Nucleic Acids Res.

[18] Huang DW, Sherman BT, Lempicki RA (2009). Systematic and integrative analysis of large gene lists using DAVID bioinformatics resources. Nat Protoc.

[19] Huang DW, Sherman BT, Lempicki RA (2009). Bioinformatics enrichment tools: Paths toward the comprehensive functional analysis of large gene lists. Nucleic Acids Res.

[20] Gramates LS, Marygold SJ, Santos GD (2017). FlyBase at 25: Looking to the future. Nucleic Acids Res.

[21] Lemaitre Lab Uplem, List of Drosophila gene potentially involved in the immune response. https://lemaitrelab.epfl.ch/page-7767-en.html.

[22] Society for Developmental Biology, the Interactive Fly: RNAi and posttranscriptional gene silencing, 2017. http://www.sdbonline.org/sites/fly/aignfam/rnaistuf.htm.

[23] Smith AM, Perelson AS (2011). Influenza A virus infection kinetics: Quantitative data and models. Wires Syst Biol Med.

[24] Read EL, Tovodwyer AA, Chakraborty AK (2012). Stochastic effects are important in intrahost HIV evolution even when viral loads are high. Proc Natl Acad Sci USA.

[25] Pearson A, Lux A, Krieger M (1995). Expression cloning of dSR-CI, a class C macrophage-specific scavenger receptor from *Drosophila melanogaster*. Proc Natl Acad Sci USA.

[26] Ligoxygakis P, Pelte N, Ji C (2002). A serpin mutant links Toll activation to melanization in the host defence of *Drosophila*. EMBO J.

[27] Gendrin M, Zaidman-Rémy A, Broderick NA (2013). Functional analysis of PGRP-LA in *Drosophila* immunity. PLoS One.

[28] Rynes J, Donohoe CD, Frommolt P (2012). Activating transcription factor 3 regulates immune and metabolic homeostasis. Mol Cell Biol.

[29] Wang H, Cai Y, Chia W (2006). *Drosophila* homologs of mammalian TNF/TNFR-related molecules regulate segregation of Miranda/Prospero in neuroblasts. EMBO J.

[30] Werner T, Liu G, Kang D (2000). A family of peptidoglycan recognition proteins in the fruit fly *Drosophila melanogaster*. Proc Natl Acad Sci USA.

[31] Tsail CW, Mcgraw EA, Ammar ED (2008). *Drosophila melanogaster* mounts a unique immune response to the Rhabdovirus *sigma virus*. Appl Environ Microb.

[32] Kim YS, Ryu JH, Han SJ (2000). Gram-negative bacteria-binding protein, a pattern recognition receptor for lipopolysaccharide and β-1,3-glucan that mediates the signaling for the induction of innate immune genes in *Drosophila melanogaster* cells. J Biol Chem.

[33] Chamy LE, Leclerc V, Caldelari I (2008). Danger signal and PAMP sensing define binary signaling pathways upstream of Toll. Nat Immunol.

[34] Watson FL, Püttmannholgado R, Thomas F (2005). Extensive diversity of Ig-superfamily proteins in the immune system of insects. Science.

[35] Zambon RA, Nandakumar M, Vakharia VN (2005). The Toll pathway is important for an antiviral response in *Drosophila*. Proc Natl Acad Sci USA.

[36] Grech A, Quinn R, Srinivasan D (2000). Complete structural characterisation of the mammalian and *Drosophila TRAF* genes: Implications for *TRAF* evolution and the role of RING finger splice variants. Mol Immunol.

[37] Cha GH, Cho KS, Lee JH (2003). Discrete functions of *TRAF1* and *TRAF2* in *Drosophila melanogaster* mediated by c-Jun N-terminal kinase and NF-κB-dependent signaling pathways. Mol Cell Biol.

[38] Berkey CD, Blow N, Watnick PI (2009). Genetic analysis of *Drosophila melanogaster* susceptibility to intestinal *Vibrio cholerae* infection. Cell Microbiol.

[39] Moy RH, Gold B, Molleston JM (2014). Antiviral autophagy restricts Rift Valley fever virus infection and is conserved from flies to mammals. Immunity.

[40] Xu YC, Wu RF, Gu Y (2002). Involvement of TRAF4 in oxidative activation of c-Jun N-terminal kinase. J Biol Chem.

[41] Ha EM, Oh CT, Bae YS (2005). A direct role for dual oxidase in *Drosophila* gut immunity. Science.

[42] Tian C, Gao B, Rodriguez MC (2008). Gene expression, antiparasitic activity, and functional evolution of the drosomycin family. Mol Immunol.

[43] Lematire B, Nicolas E, Michaut L (1996). The dorsoventral regulatory gene cassette *spätzle/Toll/cactus* controls the potent antifungal response in *Drosophila* adults. Cell.

[44] Kurucz E, Markus R, Zsamboki J (2007). Nimrod, a putative phagocytosis receptor with EGF repeats in *Drosophila* plasmatocytes. Curr Biol.

[45] Van Mierlo JT, Bronkhorst AW, Overheul GJ (2012). Convergent evolution of Argonaute-2 slicer antagonism in two distinct insect RNA viruses. PLoS Pathog.

[46] Kemp C, Imler JL (2009). Antiviral immunity in *Drosophila*. Curr Opin Immunol.

[47] Baker DA, Russell S (2009). Gene expression during *Drosophila melanogaster* egg development before and after reproductive diapause. BMC Genomics.

[48] Potempa J, Korzus E, Travis J (1994). The serpin superfamily of proteinase inhibitors: Structure, function, and regulation. J Biol Chem.

[49] Irving P, Trozler L, Heuer TS (2001). A genome-wide analysis of immune responses in *Drosophila*. Proc Natl Acad Sci USA.

[50] Tanj T, Yun EY, Ip YT (2010). Heterodimers of NF-κB transcription factors DIF and Relish regulate antimicrobial peptide genes in *Drosophila*. Proc Natl Acad Sci USA.

